# Determination and comparative analysis of 13 nucleosides and nucleobases in natural fruiting body of *Ophiocordyceps sinensis* and its substitutes

**DOI:** 10.1080/21501203.2017.1385546

**Published:** 2017-10-26

**Authors:** Wenming Cheng, Xun Zhang, Qiang Song, Weili Lu, Tingni Wu, Qunlin Zhang, Chunru Li

**Affiliations:** a School of Pharmacy, Anhui Provincial Key Laboratory of Bioactivity of Natural Product, Anhui Medical University, Hefei, Anhui, China; b Zhejiang BioAsia Institute of Life Science, Pinghu, Zhejiang, China; c Anhui Provincial Key Laboratory of Microbial Pest Control, Anhui Agricultural University, Hefei, Anhui, China

**Keywords:** *Ophiocordyceps sinensis*, nucleoside, nucleobase, high pressure liquid chromatography (HPLC), principal component analysis (PCA), cluster analysis (CA)

## Abstract

Nucleosides and nucleobases are one of the most important indicators of quality control. A sensitive and reliable high performance liquid chromatography-ultraviolet method was applied to analyse 13 nucleosides and nucleobases simultaneously in 15 batches of nine *Ophiocordyceps* species and its allies in China. Principal component analysis (PCA) and cluster analysis were conducted by SPSS 22.0 software (IBM Corp., Armonk, NY, USA). The 15 samples of *Cordyceps* were differentiated successfully based on their nucleoside and nucleobase content. Total nucleosides content in mycelium was significantly higher than that in the natural fruiting bodies of *Ophiocordyceps sinensis* (NFOS). Five nucleosides or nucleobases – adenine (A), guanosine (Gu), uracil (U), uridine (Ur) and guanine (G) – were the major components contributed to the total variance according to PCA. The profiles of the 13 tested nucleosides and nucleobases (including adenosine, cytidine, guanosine, inosine, thymidine, uridine, cordycepin, adenine, cytosine, guanine, thymine, uracil and hypoxanthine) can discriminate different samples and can be candidate indicators applied for the quality control of *Ophiocordyceps* and its allies.

## 1. Introduction

*Ophiocordyceps sinensis* (Berk.) G.H. Sung, J.M. Sung, Hywel-Jones & Spatafora, the most famous and valuable species in the genus of *Ophiocordyceps*, has been used as herbal medicine in China for centuries. Pharmacological studies on *O. sinensis* have revealed that the fungus and its fruiting bodies exerted multiple biological and pharmacological effects, such as immunomodulatory, anti-inflammatory, antioxidant, anti-ageing, antitumour, neuroprotective, hepatoprotective and renoprotective effects, and it has been applied to the treatment of kidney, liver and lung disease (Paterson 2008; Shashidhar et al. 2013). Chemical studies on the natural fruiting bodies of *Ophiocordyceps sinensis* (NFOS) have shown the presence of various carbohydrates, nucleosides, cyclodepsipeptides, alkaloids, sterols, fatty acids, amino acids, vitamins, trace minerals and other components (Lo et al. 2013; Zhao et al. 2014). Due to its medicinal uses, the non-sustainable collection of NFOS is continuously increasing, and the wild resource is decreasing rapidly. The production of NFOS cultured artificially was reported to be only feasible at laboratory scale so far, and successful cultivation for commercial purpose has not been achieved up till now (Li et al. 2006a).

For the high price of the natural fruiting bodies of *Ophiocordyceps sinensis* (NFOS) and exceeding content of some heavy metals (e.g. Pb, Hg, As, Cd) in NFOS, seeking alternatives to its goal in recent years has been the efforts of scientists. To search for the substitute of NFOS, various entomogenous fungi, mainly *Cordyceps* spp. *sensu lato*, including *C. guangdongensis, C. gunnii, C. hawkesii, C. kyushuensis, C. militaris, C. takaomontana, Elaphocordyceps ophioglossoides, O. gracilis, O. formosana, O. japonensis, O. longissima, O. sobolifera*, and some of their allies have been researched (Li et al. 2006b). These fungi related to *Cordyceps sensu lato*, although maybe not the anamorphic stage of the latter, such as *Acremonium implicatum, Mortierella hepiali*, and *Paecilomyces hepiali*, are beneficial and have been developed as health food or medicine for more than 20 years in China (Zhou et al. 2009).

Nucleosides, most of which have been isolated, are believed to be the active components in *Ophiocordyceps* species and its allies. The profile of nucleosides, nucleobases and uniquely modified nucleosides, instead of any single metabolite, can be feasible for the quality control of *Cordyceps sensu lato* products (Shiao et al. 1994; Xiao and Xiong 2013). The nucleoside profile of *Ophiocordyceps* species and its allies has been explored, and the analytical methods of determining nucleosides and nucleobases in Cordyceps have been reported previously (Li et al. 2004; Fan et al. 2006; Yang et al. 2007, 2009; Xiao and Xiong 2013; Zhang et al. 2014; Qian and Li 2017). High performance liquid chromatography (HPLC), combined with ultraviolet (UV), evaporative light scattering detector or mass spectra, has been applied to analyse the profile of nucleosides, nucleobases in *Cordyceps*-related species, and proved to be a simple, convenient and reliable method.

In this work, 15 batches of *Cordyceps* samples from nine *Ophiocordyceps-*related species, diverse sources and culture conditions were analysed by HPLC, and they were discriminated with principal component analysis (PCA) and cluster analysis. Furthermore, nucleosides and bases of several tested samples, such as *Ophiocordyceps formosana*, were firstly reported.

## 2. Materials and methods

### 2.1. Materials

Shimadzu LC-20AT HPLC system (Shimadzu, Kyoto, Japan) was equipped with a vacuum degasser, a quaternary pump, a column temperature controller, an autosampler and an SPD-20A detector.

**Figure 1.**
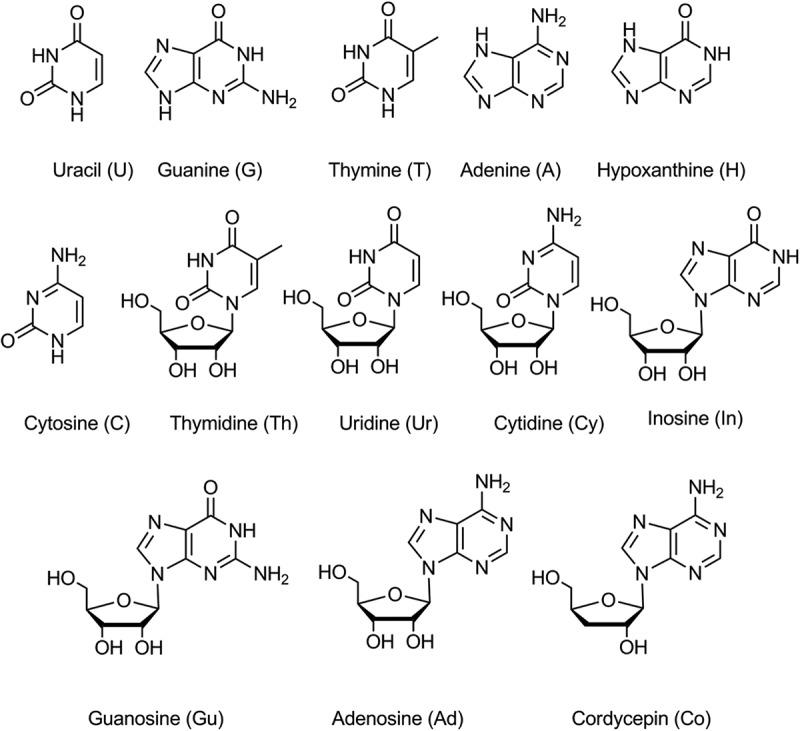
Structure of the nucleosides and nucleobases.

Standard of adenosine, cytidine, guanosine, inosine, thymidine, uridine, cordycepin and their bases adenine, cytosine, guanine, thymine, uracil and hypoxanthine (the structures are shown in Figure 1) were purchased from Sigma-Aldrich (St. Louis, MO, USA). Methanol of HPLC grade was provided by TJSHIED Company (Tianjin, China). Deionised water was prepared using a Millipore Milli-Q Plus system (Millipore, Bedford, MA, USA).

NFOS, artificially cultured *Cordyceps militaris*, the fruiting body and fermented mycelia were purchased from different sources (Table 1). All samples were identified by Professor Chunru Li and all voucher specimens were deposited at Anhui Provincial Key Laboratory of Bioactivity of Natural Product, Anhui Medical University.

**Table 1.** The sample list of *Ophiocordyceps* and its allies.

No.	Sample type	Latin name	Source
B	Artificial fruiting bodies	*Ophiocordyceps longissima*	Anhui Agricultural University
C	Cultured mycelia	*Hirsutella longissima*	Anhui Agricultural University
D	Natural fruiting bodies	*O. sinensis*	Sichuan
E	Natural fruiting bodies	*O. sinensis*	Tibet
F	Natural fruiting bodies	*O. sinensis*	Qinghai
G	Artificial fruiting bodies	*Cordyceps militaris*	Liaoning
H	Fermented mycelia	*Paecilomyces hepiali*	Wanfeng, Zhejiang
I	Fermented mycelia	*Paecilomyces hepiali*	Guoyao, Jiangxi
J	Fermented mycelia	*Cephalosporium sinensis*	Wanfeng, Zhejiang
K	Fermented mycelia	*Cephalosporium sinensis*	Zhongke, Jiangxi
L	Fermented mycelia	*Hirsutella hepiali*	Johncan, Zhejiang
M	Fermented mycelia	*Hirsutella sinensis*	Xueyu, Zhejiang
N	Sizhuang mycelia	*Acremonium implicatum*	Anhui Agricultural University
O	Fermened mycelia^a^	*O. formosana*	Anhui Agricultural University
P	Fermened mycelia^b^	*O. formosana*	Anhui Agricultural University

^a^Fermented on insect special culture medium. ^b^Fermented on SDY *culture medium*.

### 2.2. Preparation of standard and sample solutions

The standard stock solution of each analyte (~0.2 mg/mL) except guanine was prepared in initial mobile phase (0.01 mol/L potassium dihydrogen phosphate aqueous) and stored in a refrigerator. Guanine (0.2 mg/mL) was prepared in 0.1 mol/L HCl (Fan et al. 2006). The mixed standard stock solution was prepared by adding the standard stock solutions of the analytes together. A certain volume of the stock solution was transferred to a 5-mL volumetric flask and made up to volume with the same solvent to obtain the appropriate concentrations.

All samples were ground into powder and dried at 60°C. Subsequently, the precisely weighed powder (1.0 g) was mixed with approximately 20 mL of water, and then extracted ultrasonically for 1 h at room temperature (Guo et al. 2006). The extract solution was filtrated through analytical filter paper. After the residue was washed with water, the filtrate was transferred into 50-mL volumetric flask and made up to the volume. The sample was finally filtrated through a 0.45-μm membrane prior to injection into HPLC.

### 2.3. HPLC conditions

Chromatographic separations were carried out on a TSKgel ODS-100V column (250 mm × 4.6 mm id, 5 mm) operated at 20°C. The mobile phase composed 0.01 mol/L potassium dihydrogen phosphate aqueous solution (A) and methanol (B). The separation was achieved by gradient elution of 0–12 min, 0–2% B; 12–23 min, 2–5% B; 23–35 min, 5–20% B; then keeping 50% B for 10 min. A pre-equilibration period of 15 min was used between individual runs. The flow rate was kept at 1 mL/min, and the injection volume was 20 μL. The analytes were monitored for nucleosides and bases at 260 nm (Zhang et al. 2014).

### 2.4. Validation of the method

The linearity of the method was obtained by plotting peak areas of the standard compounds versus their concentrations. The standard stock solutions containing 13 reference compounds were prepared and diluted to a series of appropriate levels for the construction of calibration curves. Every calibration curve covered at least six levels. Each level was injected in triplicate, and linear regression was constructed on plots of peak areas against the concentration of each analyte. The solutions of the analytes were further diluted with mobile phase to give a series of concentrations. The limit of detection (LOD) and the limit of quantitation (LOQ) were defined at the signal-to-noise ratio (S/N) of about 3 and 10, respectively.

The intraday and inter-day variations were chosen to evaluate the precision of the method. For intraday variation test, the known concentrations of mixed standard solutions were analysed in six replicates during a single day, while for inter-day, variation test the solutions were detected in duplicates for 3 consecutive days. Five different working solutions prepared from the same sample were analysed to confirm the repeatability. One of the solutions was periodically examined at 0, 3, 6, 12, 24 h, respectively, to evaluate their stability. The relative standard deviation (RSD) was used as a measure of all the variations.

A recovery test was carried out to evaluate the accuracy of the method by adding high (120%), middle (100%) and low (80%) levels of the corresponding reference compounds into precisely weighed samples (1.0 g). Then the spiked samples were extracted and analysed in triplicates under the methods mentioned above. The average recoveries were calculated by the formula: recovery (%) = (amount found − original amount)/amount spiked × 100%.

### 2.5. Data analysis

The nucleobases and nucleosides were identified by comparing their retention time with their reference compounds. The PCA was performed using SPSS 22.0 software (IBM Corp., Armonk, NY, USA).

## 3. Result and discussion

### 3.1. Optimisation of extraction method

**Figure 2.**
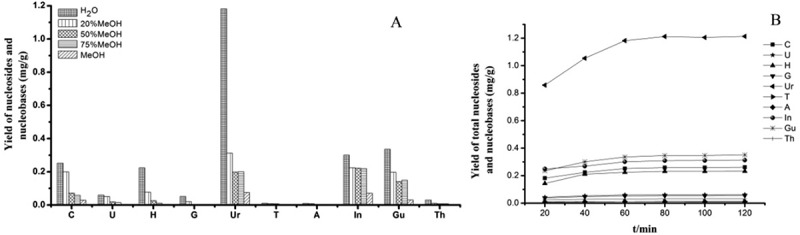
(a) Efficiencies of extraction for the analytes using different extract solvent in *O. longissima* (PS. Cytidine, cuanine and cordycepin were not detected). (b) Content of total nucleosides and nucleobases in different extract time in *O. longissima.*

Variables such as solvent, extraction method and extraction time were tested to optimise the extraction process. Nucleosides and nucleobases are hydrophilic because of their high polarity. Therefore, water and methanol are usually chosen as the extraction solvent of nucleosides and nucleobases. In this study, different proportions of aqueous methanol were tested for its efficiency as the extraction solvent. An increase of methanol had a negative influence on the extraction yield of the analytes. The best solvent was proved to be pure water that produced highest yields of nucleosides from the tested analytes. Ultrasonic extraction and reflux were selected to determine the better extraction method (Zhang et al. 2014). Both extraction methods were proved to be more efficient and faster than percolation and maceration. The results suggested that ultrasonic extraction were more effective than reflux. The extraction time was optimised using four samples in triplicate (0.2 g per sample) extracted ultrasonically with water for 20, 40, 60, 80, 100 and 120 min. It was found that the yield of the target constituents did not increase significantly after 60 min. As a result, the optimal extraction time was 60 min (Figure 2).

### 3.2. Optimisation of chromatographic condition

**Figure 3.**
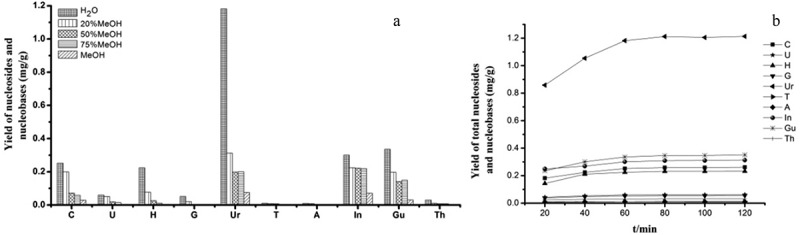
The HPLC chromatograms of mixed standards and 13 samples of different *Ophiocordyceps* species and its allies. 1. Cytosine; 2. Uracil; 3. Cytidine; 4. Hypoxanthine; 5. Guanine; 6. Uridine; 7.Thymine; 8. Adenine; 9. Inosine; 10. Guanosine; 11. Thymidine; 12. Adenosine; 13. Cordycepin. (a) Mixed nucleoside and nucleobase standards; (b) *Ophiocordyceps longissima* fruiting body; (c) *O. longissima* mycelium; (d) NFOS, Sichuan; (e) NFOS, Tibet; (f) NFOS, Qinghai; (g) *C. militaris*; (h) *Paecilomyces hepiali*, Zhejiang; (i) *Paecilomyces hepiali*, Jiangxi; (j) *Cephalosporium sinensis*, Zhejiang; (k) *Cephalosporium sinensis*, Jiangxi; (l) *Hirsutella hepiali*; (m) *Hirsutella sinensis*; (n) *Acremonium implicatum* (Sizhuang mycelia); (o) *Ophiocordyceps formosana* (mycelia on insect special medium); (p) *O. formosana* (mycelia on SDY medium).

The chromatographic condition was optimised to achieve a proper resolution of adjacent peaks. Selecting a proper chromatographic column was the first step to build an analytical method. Due to the similar molecular structures of the analytes, it was a challenge to obtain acceptable separations especially for hypoxanthine (H) and guanine (G). Several reversed-phase columns were tested to separate the 13 nucleosides and nucleobases such as TSKgel ODS 100-V, TSKgel ODS 100-Z, Shim-pack VP-ODS, Hypersil ODS2 (Dalian Elite, China). The results showed that the TSKgel ODS 100-V column could separate H and G with better resolution than the other columns. Another reason for the selection of TSKgel ODS 100-V column is that it can be used in 100% aqueous solution. Two organic solvents (methanol and acetonitrile) and three kinds of the aqueous solutions (2% Acetic acid aqueous solution, 0.3% phosphoric acid aqueous solution and 0.01 mol/L potassium dihydrogen phosphate aqueous solution) were examined to optimise the mobile phase. The result revealed that methanol–0.01 mol/L KH_2_PO_4_ aqueous solution system exhibited better separation than other tested systems. The initial mobile phase was 100% aqueous solution, and gradient elution was employed because the analytes have different polarities. The wavelength of maximum absorption of 13 nucleosides and nucleobases were observed at 260 nm (Zhang et al. 2014). The investigated compounds were identified by comparison of their retention times and their UV spectra under the same condition. The HPLC chromatograms of standards of 13 nucleosides and nucleobases and different *Ophiocordyceps* species and its allies are shown in Figure 3.

### 3.3. Method validation

The validation of the proposed method for quantitative analysis was determined by the linearity, LOD, LOQ, intraday and inter-day precisions, stability, repeatability and accuracy. The correlation coefficient values (*r*) were more than 0.9990 for all the analytes, demonstrating good linearity between the investigated compounds’ concentrations and their peak areas within the test ranges. The LODs and LOQs for the 13 nucleosides and nucleobases were less than 0.137 and 0.452 μg/mL, respectively, as shown in Table 2. The overall intraday and inter-day variations (RSDs) were less than 2.0% (*n* = 5) for all analytes. The overall recoveries were in the range from 98.38% to 107.47% (*n* = 5) with RSDs less than 3.69% for the analytes. The results showed that the HPLC method was sensitive, repeatable and accurate for the simultaneous determination of the 13 nucleosides and nucleobases.

**Table 2.** Calibration curves, LOD and LOQ for the nucleoside and nucleobase standards.

Analyte	Calibration curve^a^	*r*^b^	Liner ranger (μg/mL)	LOD^c^ (μg/mL)	LOQ (μg/mL)
Cytosine	*Y* = 52196*X* + 3589.4	0.9997	0.15–15.15	0.030	0.099
Uracil	*Y* = 108810*X* + 8397.2	0.9997	0.15–15.46	0.016	0.053
Cytidine	*Y* = 33596*X* + 2939.9	0.9998	0.15–15.38	0.063	0.208
Hypoxanthine	*Y* = 72926*X* + 5192.4	0.9996	0.17–16.85	0.137	0.452
Guanine	*Y* = 40114*X* + 2428.9	0.9994	0.15–15.31	0.124	0.409
Uridine	*Y* = 41926*X* + 2806.4	0.9998	0.16–15.85	0.064	0.211
Thymine	*Y* = 64016*X* + 5207.2	0.9996	0.15–15.08	0.061	0.201
Adenine	*Y* = 100851*X* + 5796.1	0.9997	0.15–15.00	0.122	0.403
Inosine	*Y* = 29236*X* + 3077.7	0.9993	0.17–16.54	0.067	0.221
Guanosine	*Y* = 39089*X* + 2761.7	0.9995	0.16–15.85	0.064	0.211
Thymidine	*Y* = 42089*X* + 2851.1	0.9995	0.16–15.69	0.064	0.208
Adenosine	*Y* = 62169*X* + 6009.6	0.9990	0.16–15.54	0.032	0.106
Cordycepin	*Y* = 62071X + 6874.8	0.9990	0.16–16.08	0.033	0.109

^a^The calibration curves were constructed on plots the peak areas versus the concentration of each analyte. Each calibration curve included eight data points. ^b^*r* Refers to correlation coefficient. ^c^LOD refers to the limit of detection. LOQ refers to the limit of quantification.

### 3.4. Quantification of nucleosides and nucleobases in *Ophiocordyceps* species and its allies

The developed HPLC method was subsequently applied to determine the profiles of 13 nucleosides and nucleobases in 15 batches of samples from nine different *Cordyceps sensu lato* species simultaneously. The results of the quantitative analysis are shown in Table 3. Total contents of nucleoside and nucleobase ranged from 2.46 to 13.38 mg/g. The contents of nucleosides and nucleobases were unbalanced in different species and species from various habitats and specific culture conditions.

**Table 3.** The contents (mg/g) of 13 nucleosides and nucleobases in different *Ophiocordyceps* and its allies.

No.	C	U	Cy	H	G	Ur	T	A	In	Gu	Th	Ad	Co	Total
B	0.25	0.06	−^a^	0.22	0.052	1.18	0.01	0.01	0.30	0.34	0.03	+^b^	−	2.46
C	0.017	0.56	−	0.004	−	0.97	0.01	0.053	0.39	2.11	0.04	1.50	−	5.65
D	0.044	0.01	−	0.026	0.038	1.80	−	0.017	1.22	1.24	0.05	0.11	−	4.54
E	0.043	0.005	0.004	0.029	−	1.83	−	0.018	1.26	1.32	0.08	0.25	0.02	4.85
F	0.015	0.039	0.005	0.10	−	1.68	−	0.018	0.90	0.71	0.041	0.007	−	3.51
G	0.019	0.013	0.001	0.002	−	2.92	−	0.026	0.19	0.90	0.33	1.70	0.84	6.94
H	0.067	0.085	0.39	0.007	0.35	1.98	−	0.33	0.13	2.54	0.13	2.13	0.024	8.17
I	0.075	0.07	0.97	−	0.44	4.02	0.004	0.45	0.075	3.38	0.03	2.74	−	12.25
J	0.035	0.83	0.078	0.05	0.97	4.94	0.005	0.78	0.087	2.81	0.13	2.66	−	13.38
K	0.040	0.658	0.148	0.031	0.58	4.56	0.022	0.60	0.092	3.07	0.10	2.59	0.004	12.49
L	+	0.074	0.100	0.18	0.079	0.47	0.005	0.09	1.63	0.18	0.032	2.81	−	5.64
M	0.01	0.11	0.30	0.005	0.099	5.19	0.017	0.37	0.19	3.74	0.13	2.79	−	12.95
N	0.005	0.521	0.009	0.45	+	5.27	0.13	0.49	1.43	3.48	0.27	0.95	−	13.00
O	0.005	+	−	−	0.002	0.33	0.02	0.006	0.89	0.16	+	0.75	0.005	2.16
P	0.021	+	0.036	−	−	2.68	0.006	0.041	0.16	2.31	0.032	2.003	0.021	7.29

^a^− Not detected. ^b^+ Below LOQ.

The fermented samples, including sample J (C*ephalosporium sinensis*), M (*Hirsutella sinensis*), N (*Acremonium implicatum*), K (*Cephalosporium sinensis*) and I (*Paecilomyces hepiali*), contained a relatively higher level of total nucleosides. Uridine, guanosine and adenosine were the major components of the most tested samples.

Adenosine, which plays an important role of biochemical process in organism and is a major nucleoside in *Ophiocordyceps* species and its allies, was found with higher content in all cultivated samples than those of NFOS. Uridine was abundant in all samples except in sample L (*Hirsutella hepiali*) and O (*O. formosana*), while guanosine was less in sample L, O and B (*O. longissima*). The levels of cytidine and uracil varied considerably in different *Cordyceps sensu lato* and its allies. Inosine is higher in sample L, N and NFOS. Inosine is applicable to leukopenia, thrombocytopenia, various heart diseases, acute and chronic hepatitis, cirrhosis, etc., in addition to its fair treatment of retinitis and optic nerve atrophy (Haskó et al. 2004).

Cytidine was rich in sample I but very little in other species. Uracil existed in a higher level in sample J, K, C (*O. longissima*) and N than the other samples. Thymine and cordycepin were found in small quantities or even not detected, except the fact that cordycepin is relatively higher in sample G (*Cordyceps militaris*) (0.84 mg/g), lower than the data reported by Ikeda et al. (2008) (4.85 mg/g), and by Wang et al. (2009) (18.22 mg/g in CM-51762), while higher than that by Wang et al. (2009) (0.05 mg/g in CMM-ZJG). The high content of cordycepin has been also reported in *Cordyceps kyushuensis* (1.42, 2.54 mg/g in the natural fruiting body and cultured fruiting body, respectively) (Ling et al. 2002), implying a very close species to *Cordyceps militaris*.

The content value of nucleosides and nucleobases is affected by different factors, such as culture medium, temperature, time and sample preparation method, which explain the difference of data in the same species of various origin and diverse culture conditions (Li et al. 2001; Yu et al. 2006; Zhao et al. 2014).

### 3.5. PCA of samples

**Figure 4.**
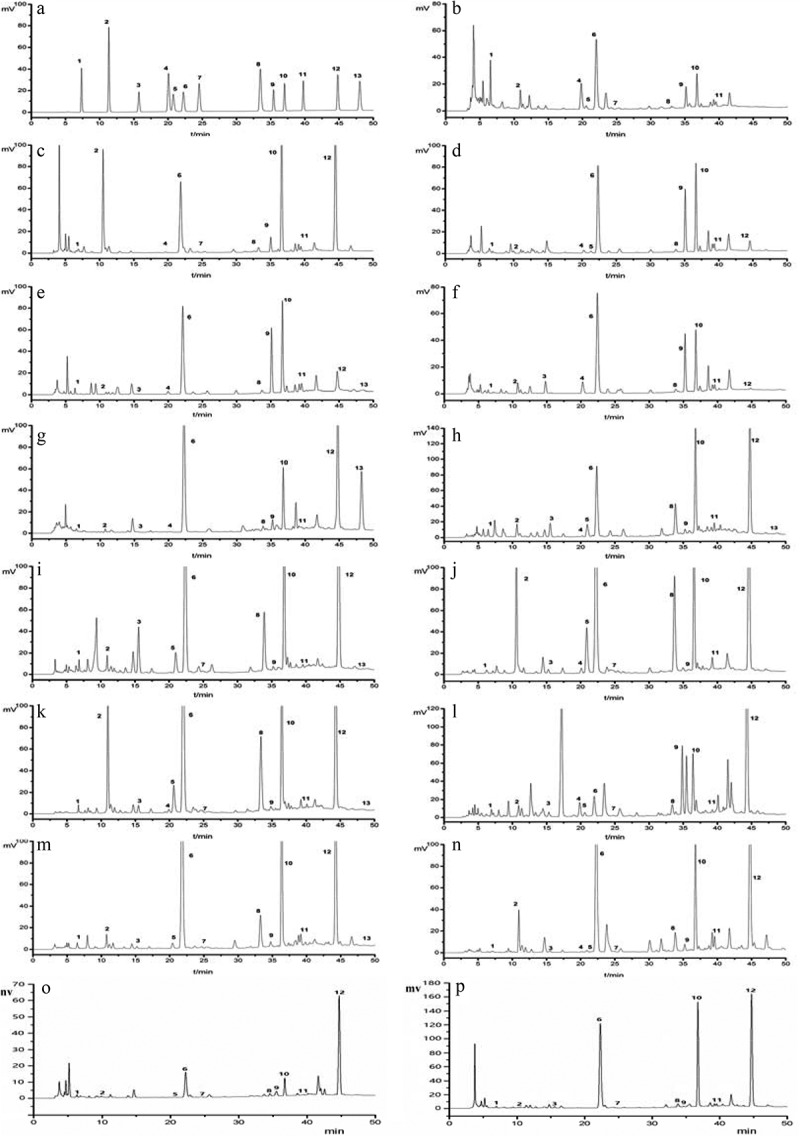
PCA loading plots derived from 13 nucleosides and nucleobases of 15 Cordyceps samples.

**Figure 5.**
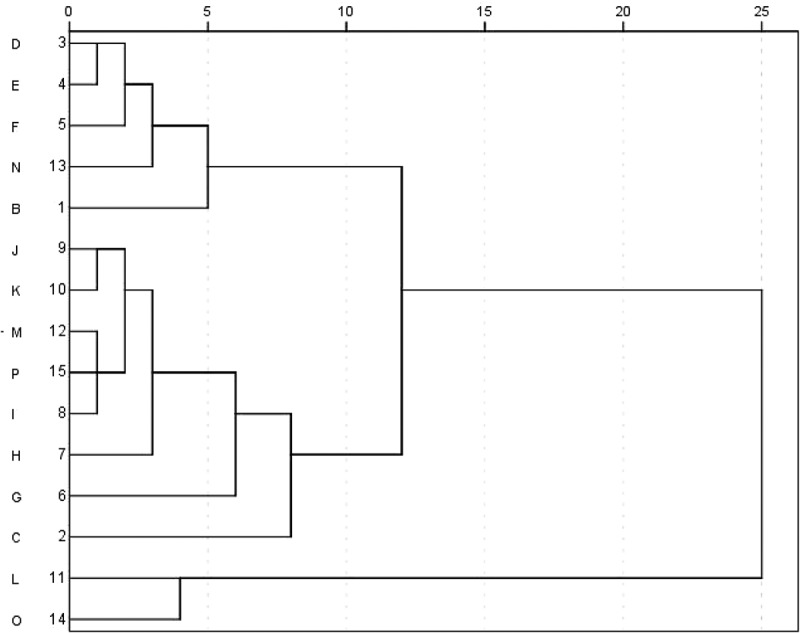
CA analysis based on the 13 nucleosides and nucleobases of 15 Cordyceps samples.

To further evaluate the variation of nucleosides and nucleobases in all samples, PCA was performed on the basis of the contents of 13 tested compounds from HPLC profiles by SPSS 22.0 for windows. The first three principal components (PC 1, PC 2, PC 3) with more than 72.55% of the total variance were extracted for analysis. Among them, PC1 accounted for 37.12% of the whole variance, whereas PC2 explained 21.33% of the total variance, respectively. The scatter plot is shown in Figure 4, which shows adenine (A), guanosine (Gu), uracil (U), uridine (Ur) and guanine (G) were the major components contributed to the total variance. The distribution of nucleosides and nucleobases in all the samples showed the difference in the origin, species and culture condition difference (Figure 5). The NFOS samples were clustered together, but they were far from sample L and O. This indicated that NFOS could be preliminarily discriminated from sample L and O by the profiles of nucleosides and nucleobases. Except for sample L, O, C, G and B, the other tested samples were close together, which demonstrated that they were similar in the profiles of the tested nucleosides and nucleobases.

## 4. Conclusion

In this study, a simple and reliable method has been developed for simultaneous analysis of 13 nucleosides and nucleobases in 15 batches of samples from nine *Ophiocordyceps* species and its allies. All tested samples were determined and distinguished by this method.

Total nucleosides content in mycelium was significantly higher than that in the fruiting body. The profiles of the tested nucleosides and nucleobases in NFOS and its allies were different due to the diverse sources, various species and certain culture conditions. Among various species, the fermented samples, including sample J (C*ephalosporium sinensis*), M (*Hirsutella sinensis*), N (*Acremonium implicatum*), K (*Cephalosporium sinensis*) and I (*Paecilomyces hepiali*), have higher levels of nucleosides. Five nucleosides or nucleobases – adenine (A), guanosine (Gu), uracil (U), uridine (Ur) and guanine (G) – were the major components contributed to the total variance according to PCA. The content of cordycepin seems to be useful markers for distinction of certain species. The profiles of the tested nucleosides and nucleobases can discriminate different samples and can be candidate indicators applied for the quality control of *Ophiocordyceps* and its allies.
